# An international comparative analysis and roadmap to sustainable biosimilar markets

**DOI:** 10.3389/fphar.2023.1188368

**Published:** 2023-08-24

**Authors:** Khalid A. Alnaqbi, Agnès Bellanger, Alex Brill, Gilberto Castañeda-Hernández, Ana Clopés Estela, Olga Delgado Sánchez, Pilar García-Alfonso, Pius Gyger, Daniel Heinrich, Germain Hezard, Adriana Kakehasi, Cheryl Koehn, Olivier Mariotte, Francesco Mennini, Sonia Mayra Pérez-Tapia, Michele Pistollato, Rowan Saada, Tadanori Sasaki, George Tambassis, Marc Thill, Gustavo Werutsky, Tim Wilsdon, Steven Simoens

**Affiliations:** ^1^ Tawam Hospital, College of Medicine and Health Sciences, UAE University, Al Ain, United Arab Emirates; ^2^ Pitié Salpétrière Hospital, Sorbonne University, Paris, France; ^3^ Matrix Global Advisors, American Enterprise Institute, Washington, DC, United States; ^4^ Centro de Investigación y de Estudios Avanzados del Instituto Politécnico Nacional, Mexico City, Mexico; ^5^ Catalan Institute of Oncology, Barcelona, Spain; ^6^ Espases University Hospital, Palma de Mallorca, Spain; ^7^ Medical Oncology Department, Gregorio Marañón General University Hospital, Instituto de Investigación Sanitaria Gregorio Marañón (IiSGM), Universidad Complutense, Madrid, Spain; ^8^ Independent Consultant, Zurich, Switzerland; ^9^ Innlandet Hospital Trust, Gjøvik, Norway; ^10^ Nile, Paris, France; ^11^ Federal University of Minas Gerais (UFMG), Belo Horizonte, Brazil; ^12^ Arthritis Consumer Experts, Vancouver, BC, Canada; ^13^ Economic Evaluation and HTA (EEHTA), CEIS, Faculty of Economics, University of Rome “Tor Vergata”, Rome, Italy; ^14^ Unidad de Investigacion, Desarollo e Innovacion Médica y Biotecnológica (UDIMEB), Unidad de Desarollo e Investigacion de Bioterapeuticos (UDIBI), Instituto Politécnico Nacional, Ciudad de México, Mexico; ^15^ Charles River Associates, London, United Kingdom; ^16^ Showa University, Shinagawa, Japan; ^17^ Australian Biosimilars Academy, Sydney, NSW, Australia; ^18^ Department of Gynecology and Gynecological Oncology, Agaplesion Markus Hospital, Frankfurt, Germany; ^19^ Hospital São Lucas PUCRS, Porto Alegre, Brazil; ^20^ KU Leuven, Leuven, Belgium

**Keywords:** biosimilar, sustainability, policy, market access, comparative analysis

## Abstract

**Background:** Although biosimilar uptake has increased (at a variable pace) in many countries, there have been recent concerns about the long-term sustainability of biosimilar markets. The aim of this manuscript is to assess the sustainability of policies across the biosimilar life cycle in selected countries with a view to propose recommendations for supporting biosimilar sustainability.

**Methods:** The study conducted a comparative analysis across 17 countries from North America, South America, Asia-Pacific, Europe and the Gulf Cooperation Council. Biosimilar policies were identified and their sustainability was assessed based on country-specific reviews of the scientific and grey literature, validation by industry experts and 23 international and local non-industry experts, and two advisory board meetings with these non-industry experts.

**Results:** Given that European countries tend to have more experience with biosimilars and more developed policy frameworks, they generally have higher sustainability scores than the other selected countries. Existing approaches to biosimilar manufacturing and R&D, policies guaranteeing safe and high-quality biosimilars, exemption from the requirement to apply health technology assessment to biosimilars, and initiatives counteracting biosimilar misconceptions are considered sustainable. However, biosimilar contracting approaches, biosimilar education and understanding can be ameliorated in all selected countries. Also, similar policies are sometimes perceived to be sustainable in some markets, but not in others. More generally, the sustainability of the biosimilar landscape depends on the nature of the healthcare system and existing pharmaceutical market access policies, the experience with biosimilar use and policies. This suggests that a general biosimilar policy toolkit that ensures sustainability does not exist, but varies from country to country.

**Conclusion:** This study proposes a set of elements that should underpin sustainable biosimilar policy development over time in a country. At first, biosimilar policies should guarantee the safety and quality of biosimilars, healthy levels of supply and a level of cost savings. As a country gains experience with biosimilars, policies need to optimise uptake and combat any misconceptions about biosimilars. Finally, a country should implement biosimilar policies that foster competition, expand treatment options and ensure a sustainable market environment.

## 1 Introduction

A biosimilar is a biological medicine which has been shown not to have any clinically meaningful differences from the originator medicine in terms of quality, safety and efficacy (Kirchhoff et al., 2017). The manufacture of biologics from living organisms subjects them to various modifications intrinsic to biology and nature. The first regulatory pathway for biosimilars to be commercialised was established in 2005 in Europe and the first European biosimilar was approved in 2006. As of June 2023, the European Medicines Agency has approved 94 biosimilars (European Medicines Agency, 2023). In the United States, a biosimilars pathway was created in 2010 and the first United States biosimilar was approved in 2015. As of May 2023, the United States Food and Drug Administration has approved 41 biosimilars (Food and Drug Administration, 2023). Other countries also have official biosimilar guidelines, including Canada (Health Canada, 2022), Japan (Arato, 2016) and Saudi Arabia (Biomapas, 2022).

The biosimilar pipeline in different countries will continue to evolve as biologics in new therapeutic areas approach patent expiry. Biologics expected to lose exclusivity in the European Union and the United States in the next 5 years include golimumab for musculoskeletal disorders in 2024 and belimumab for musculoskeletal and haematological disorders in 2026. Although the number of biologics facing loss of exclusivity is continuing to grow, it cannot be expected that there will necessarily be biosimilars manufactured for all of these biologic products (especially within orphan indications) (Global data, 2022). Furthermore, not all biosimilars approved by the European Medicines Agency are available in the majority of European countries. This points to the existence of potential sustainability limitations across existing policy frameworks.

Many publications regarding the off-patent biological and biosimilar market refer to the importance of sustainability with regards to policy development and application. While many do not define sustainability explicitly, concepts common across publications include: “balance between incentives for all key stakeholders/multi-stakeholder benefits” (Kumar, 2018; Simoens et al., 2018; van den Hoven, 2017; Vulto et al., 2019), “cost savings/sustainability for budgets” (van den Hoven, 2017; Medicines for Europe, 2020a; Simoens and Vulto, 2021), “sustainable price competition” (GfK Market Access, 2014), “increased/broader patient access” (Dutta et al., 2020), “sustained innovation” (Allens in partnership with Linklaters LLP, 2016a; Dutta et al., 2020; Association for Accessible Medicines, 2021), “increased levels of competition and choice” (Dutta et al., 2020; IGBA, 2020; Barbier et al., 2021a), “attractiveness for continued investment” (Allens in partnership with Linklaters LLP, 2016b), and “sustainable supply” (Vulto et al., 2019; Association for Accessible Medicines, 2021). Therefore, although there is no consensus on how to define sustainability (IQVIA Institute for Human Data Science, 2018; Pugatch Consilium, 2019), a sustainable biosimilar market could be defined as an environment where “all stakeholders, including patients, benefit from appropriate and reliable access to biological therapies. Competition leads to a long-term predictable price level, without compromising quality, while delivering savings that may be reinvested” (Vulto et al., 2020).

There are multiple reasons why there is a need to understand how policy can improve sustainability of biosimilar markets. First, there is variation in biosimilar uptake between countries. As of 2021, biosimilars capture 10% of the total biologic pool in Europe, with 7% having been achieved in the last 5 years (PharmaTimes, 2021). However, biosimilar uptake in countries such as Japan, Canada and the United States has been significantly slower, although this can vary across products. This suggests that the potential for biosimilars has not been completely realised in all countries (Vogler et al., 2021). Second, the issue of lack of market confidence persists. Misconceptions around the safety of biosimilars as non-identical molecular entities result in mistrust among physicians and patients that can affect usage (Barbier et al., 2021b). Third, policies applied to biosimilars in some countries have been questioned, leading to debates (and sometimes legal battles) between payers/governments and manufacturers (Moorkens et al., 2017). For instance, inconsistent contracting practices for biosimilars in Mexico have led to a lack of stable and transparent procurement methods (Martínez, 2020). Fourth, decision makers in some countries continue to apply policies designed for generic small molecules to biosimilars. There is a concern that such policies are not tailored to the specific characteristics and market dynamics of biosimilars and, thus, create an unsustainable environment.

The aim of this study is to provide a biosimilar sustainability roadmap for 17 selected countries worldwide. To this effect, the study: a) lists and assesses the sustainability of specific biosimilar policies per country; b) conducts a sustainability rating per key biosimilar policy area per country and reports examples of best practices; c) attempts to identify a general biosimilar policy toolkit that ensures sustainability; and d) provides recommendations for biosimilar sustainability per key policy area. This comparative study wishes to learn from the global experience with biosimilar policies and inform decision makers on how to make their policy frameworks fit-for-purpose to ensure a sustainable environment for biosimilar products in the long term.

## 2 Materials and methods

### 2.1 Selection of countries

Given the global focus of the study, a wide geographic scope was adopted by including 17 countries from North America (i.e., Canada, Mexico, United States), South America (i.e., Brazil), Asia-Pacific (i.e., Australia, Japan), Europe (i.e., Belgium, France, Germany, Italy, Netherlands, Norway, Spain, Switzerland, United Kingdom) and the Gulf Cooperation Council (i.e., Saudi Arabia, United Arab Emirates). This selection was made to ensure coverage of countries in different stages of development with regards to biosimilar uptake and policies, countries from different policy archetypes (e.g., public insurance vs. strong presence of the private sector; wide use of tendering procedures vs. direct contracting agreements), countries with different levels of economic development. European countries dominated the sample as these countries have the longest experience with biosimilars.

### 2.2 Study framework

In order to identify and assess the sustainability of biosimilar policies, the research team developed a framework which distinguishes between nine key policy areas across the biosimilar life cycle (starting with manufacturing and then regulatory approval considerations through to pricing and reimbursement measures before going through prescribing and dispensing practices and ending with considerations on the monitoring of biosimilar products). The framework also used a rating system to evaluate the level of sustainability of biosimilar policies using five answer categories (i.e., “sustainable for all stakeholders,” “minor areas for sustainability improvement,” “major areas for sustainability improvement,” “presence of unsustainable policies,” “unsustainable policy environment for majority of stakeholders”).

This study used a multi-method approach, consisting of a review of the literature, expert validation and advisory board meetings.

### 2.3 Literature review and expert validation

Identification of biosimilar policies in place in a country and their sustainability assessment was performed via country-specific literature reviews and was validated with country experts. The review covered peer-reviewed articles and the grey literature (e.g., governmental official sources (e.g., legislation and health policy plans), media reports and formal white papers from national/pan-national/international regulatory authorities). Search engines included PubMed, Google Scholar and local Google sites. The following search terms were used: “(country),” “biosimilar,” “policy,” “manufacturing,” “regulatory assessment,” “health technology assessment,” “reimbursement,” “pricing,” “price discounts,” “launch pricing,” “contracting,” “tendering,” “provision,” “prescribing,” “prescribing incentives,” “prescribing practices,” “switching,” “dispensing,” “substitution,” “education,” “campaigns,” “misconceptions,” “monitoring,” and “pharmacovigilance.” The literature review focused on documents published between 2014 and 2021 written in English or local languages.

Findings from the literature review were then validated with industry experts and 23 international and local non-industry experts. One to three biosimilar non-industry experts in each country were selected. Although the number of validators per country was limited, these were chosen based on their previous publication history, contribution to biosimilar policy and level of expertise. Across countries, non-industry experts with different backgrounds were selected to ensure a range across patient representatives, physicians, pharmacists, payers and/or health economists.

### 2.4 Advisory board meetings

Informed by the comparison of the country policy landscapes, two international advisory board meetings were held online in Fall-Winter 2021 during which the non-industry experts discussed the key areas for biosimilar policy improvement across countries. Outputs from these meetings were synthesised into recommendations for each of the nine key biosimilar policy areas. These recommendations were reviewed offline by attendees to ensure alignment across stakeholders.

## 3 Results

### 3.1 Biosimilar policy landscape and sustainability assessment

Within each key biosimilar policy area, an overview of specific measures in place in each selected country in 2021 has been collected (see Table 1) and their sustainability has been assessed. This section presents the (dis)advantages of specific measures per key policy area from a sustainability perspective based on the experience of the selected countries.

**TABLE 1:** Biosimilar policy landscape in selected countries in 2021.

Policy	AUS	BEL	BRA	CAN	FRA	DEU	GBR	ITA	JPN	MEX	NLD	NOR	SAU	ESP	CHE	UAE	United States
Manufacturing and R&D																	
- Local manufacturing incentives			X											X	X		
- Early biosimilar manufacturing license		X			X	X	X	X			X	X		X	X	X	
Regulatory approval																	
- Streamlined evidence requirements	X	X	X	X	X	X	X	X	X	X	X	X	X	X	X	X	X
- Simplified regulatory approval through international collaboration										X							
- Regulatory support for biosimilar submission																	X
Health technology assessment																	
- Lack of full-length health technology assessment requirements for biosimilars			X	X		X		X	X	X	X	X	X	X	X	X	X
- Simplified health technology assessment submission requirements	X				X												
Pricing																	
- At launch—mandated fixed discounts for biosimilars	X				X			X	X					X	X	X	
- At launch—mandated fixed discounts for originators	X	X			X	X											X
- At launch—tiered discounts for biosimilars													X				
- Over time—progressive price discounts	X							X			X						
- At launch—reference pricing mechanisms	X	X	X			X	X	X			X	X		X			X
Reimbursement																	
- Automatic reimbursement following regulatory approval and submission					X	X	X	X			X	X		X		X	X
- Full versus partial coverage		X		X		X		X			X				X	X	
- Exclusionary contracts																	X
Contracting																	
- Direct contracting with providers	X	X	X	X									X		X	X	X
- Tendering practices	X	X	X		X	X	X	X		X	X	X	X	X	X	X	
Biosimilar education and understanding																	
- Healthcare professional educational programs	X	X		X	X	X	X	X			X	X	X	X	X		X
- Patient educational programs	X	X		X	X		X	X			X	X		X			X
Prescribing																	
- Clinical recommendations for prescriber-initiated biosimilar prescription	X				X	X	X	X			X		X	X	X	X	
- Mandated switching to cheapest alternative				X								X					
- Prescription quotas for volume of biosimilar prescription		X			X	X	X	X						X			
- Financial incentives linked to volume of biosimilar prescription		X			X	X	X	X	X								
- Financial penalties linked to volume of biosimilar prescription						X											
- De facto prescribing by international non-proprietary name	X									X				X	X		
Dispensing																	
- Automatic substitution	X				X						X						X
- Regressive pharmacist markups	X	X			X		X				X				X	X	X
- Reduced patient co-payments									X								
Monitoring																	
- Post-commercialisation pharmacovigilance measures	X	X	X	X	X	X	X	X	X		X	X	X	X	X	X	X
- Supply and usage monitoring	X						X				X						

Note: “X” = policy applied in the country.

#### 3.1.1 Manufacturing and R&D

Manufacturing and R&D measures applied in the selected countries include local manufacturing incentives and early biosimilar manufacturing licenses (ahead of originator loss of exclusivities) (see Table 1). Local manufacturing incentives are an instrument to boost a country’s economy and biotechnological industry, and reduce dependencies on import (Bellodi et al., 2020). Switzerland, for instance, has included local production of biosimilars in the criteria for awarding tenders (Medicines for Europe, 2020b). However, to establish sufficient local manufacturing, incentives may be required to support a national manufacturing industry that is competitive against internationally established manufacturers. Nevertheless, implementation of such incentives is limited in most countries, and international supply chains provide an alternative approach which can maintain market competition provided that sustainable criteria are used for contracts. Policies encouraging foreign manufacturing solely on the basis of price can discourage certain manufacturers to commercialise their products in the country, reducing competition and limiting cost savings and treatment options for patients.

Early biosimilar manufacturing licenses ensure that originators benefit from their full exclusivity period, while simultaneously fast and efficient access for biosimilars is facilitated. Manufacturing waivers could provide advantages in some markets, but they need to be designed with care taking into account market circumstances so that they are not anti-competitive and in conflict with free-trade agreements with countries that do not allow for such waivers.

#### 3.1.2 Regulatory approval

With respect to biosimilar regulatory approval, our sample of countries have implemented streamlined evidence requirements, simplified regulatory approval procedures through international collaboration or provide regulatory support for biosimilar submission (see Table 1). For instance, the United Kingdom Medicines and Healthcare products Regulatory Agency streamlined its evidence requirements based on the scientific state of the art and real-world experience, and announced a new approach to biosimilar approval which does not require *in vivo* studies in animals and comparative clinical trial requirements have been changed in most cases (Gov.uk, 2020). Among the selected countries, the United States Food and Drug Administration is the only regulatory agency that has established a specific support program (i.e., the Biosimilar Product Development Program) in which biosimilar manufacturers receive product-specific advice to support them to meet regulatory requirements (US Food and Drug Administration, 2018).

#### 3.1.3 Health technology assessment

Most selected countries do not require a health technology assessment for a biosimilar or have simplified health technology assessment submission requirements (see Table 1). With respect to the former, the Canadian Agency for Drugs and Technologies in Health, for example, reviewed its biosimilar procedures introduced in 2018 and concluded that a health technology assessment is not essential and delays access to biosimilars (Ascef et al., 2020). With respect to the latter, the Pharmaceutical Benefit Advisory Committee in Australia, for instance, allows for a “Category 3” submission for a biosimilar that does not apply for indications beyond those of the originator, implying that the assessment is limited to a review of clinical need and effectiveness and does not include an economic evaluation (Australian Government Department of Health, 2021).

#### 3.1.4 Pricing and reimbursement

Approaches to price setting range from free-pricing policies to pre-agreed price discounts for biosimilars (and sometimes originators) at launch or over time (see Table 1). It is critical that pricing policies are mindful of a long-term sustainable environment for all stakeholders. For example, pre-agreed price discounts can be an effective way to guarantee minimum savings to payers in smaller markets (e.g., Saudi Arabia) with lower expected levels of competition and to ensure predictability for manufacturers. However, mandatory price reductions provide little incentive for further price competition. Also, without volume guarantees for biosimilar products, aggressive price reduction policies could result in unsustainable price levels (Simon Kucher and Partners, 2016) and lead to manufacturers’ withdrawal from the market. Pricing based on market dynamics allows for differentiation between therapy areas, and can incentivise both competition and sustainable levels of cost savings.

To accelerate patient access, automatic reimbursement for biosimilars is favoured in most countries. However, there should be a transparent regulatory process regarding upcoming products to provide predictability for biosimilar and originator manufacturers and inform their supply decisions. This is an issue in countries such as Australia and Mexico, where these processes are currently opaque.

While policies limiting the level of reimbursement to the cheapest alternative can drive considerable savings, this can encourage prescribing that is not in line with medical rationale and restrict physician/patient choice. Further, continuous changes in reimbursement levels upon market entry of cheaper alternatives can affect patients already initiated on a treatment and prompt frequent switches, as well as affecting demand for marketed products, which could lead to over-supply or shortages.

Exclusionary contracts are made between originator manufacturers or manufacturers of first-to-market biosimilars and payers that can inhibit the subsequent reimbursement of other biosimilar products. For example, in the United States, some commercial plans require patients to receive access to originator products prior to a biosimilar (Mehr, 2018; Chambers et al., 2020). The implementation of exclusionary contracts might be beneficial for certain manufacturers, but reduces the potential for biosimilar success, thus decreasing competition in the long term and negating savings. This may also have negative impacts on the attractiveness of the market for future biosimilars in other therapeutic areas.

#### 3.1.5 Contracting

Direct contracting with providers is an established practice in the United States and Brazil (Smeeding et al., 2019) (see Table 1). Such contracting can happen at national or regional level. While national contracts can provide more unified treatment alternatives for healthcare professionals and patients, they might reduce competition if the number of selected suppliers is not enough. Regional contracts can ensure multiple suppliers within any given country but can lead to increased administrative burden, access delays and more complex monitoring and regulation efforts.

The sustainability of tendering practices depends on, amongst other things, the number of winners allowed, the criteria used for their awarding, and the length of the awarded contracts (Barbier et al., 2021a). The experience of the selected countries indicates that the selection of a single supplier is unlikely to be sustainable as it can disincentivise participation (Marton, 2020). Also, Norway, for example, encountered supply problems when tenders have been awarded to only one pharmaceutical company, which in the end struggled to provide enough product (IHS Markit, 2018). The inclusion of award criteria beyond price (e.g., the availability of value-added services) can encourage increased competition and provide multi-stakeholder benefits. However, monitoring of the correct use of criteria is needed. For instance, poor enforcement of a law requiring the consideration of additional criteria in Italy has led regional authorities still to apply price as the main decision factor. Finally, debates on the adequate duration of tender contracts need to consider not only opportunities to boost competition but also potential harms. For example, shorter contracts can mean frequent switching of patients’ treatments, which is not sustainable in the longer term (Hagen, 2021).

#### 3.1.6 Biosimilar education and understanding

The concept of biosimilars is fairly new for the majority of selected countries and has only affected a limited number of therapeutic areas. Therefore, the implementation of educational campaigns for healthcare professionals and patients can improve their perception about biosimilars and provide a way to promote their uptake, especially in the outpatient sector. In addition to educational campaigns (see Table 1), certain countries like the United Arab Emirates have implemented policies to counteract misconceptions and to avoid preferential treatment of originators from healthcare professionals (Sochacki, 2018). Moreover, for countries where access to biosimilars is decided through contracting (e.g., Spain and Italy), educational efforts focused on payers and other decision makers is critical as these decisions often provide little additional flexibility for physicians and pharmacists with regards to prescription and/or dispensation.

#### 3.1.7 Prescribing

Table 1 shows that the selected countries have implemented a variety of prescribing policies which promote or mandate biosimilar usage through formal/informal recommendations or incentives/penalties. Recommendations to prescribe biosimilars based on their cost-effectiveness are largely a sustainable practice that can support wider use of biosimilars. However, in some countries, such measures may not be sufficient and, therefore, the introduction of temporary interventional policy may be required to stimulate biosimilar use. A mandated switch to the lowest-cost alternative [as observed in some Canadian provinces (Canadian Society of Intestinal Research, 2021)] might drive biosimilar uptake, but it sets the grounds for diminishing competition opportunities for other stakeholders. Moreover, this practice can result in frequent switching as prices decrease, promoting a “race-to-the-bottom” and leading to unsustainable price levels. Prescribing quotas [as implemented in, for example, Germany (Moorkens et al., 2020a) and the United Kingdom (Moorkens et al., 2021)] and financial incentives or penalties linked to biosimilar prescription can boost biosimilar uptake in the short term, but do not foster natural competition between originator and biosimilars. Finally, prescribing by international non-proprietary name may support biosimilar use if accompanied by other policy measures that favour biosimilars.

#### 3.1.8 Dispensing

Biosimilar policies regarding dispensing in the selected countries relate to substitution and financial incentives for pharmacists and patients (see Table 1). Automatic substitution without input from both prescribing physician and dispensing pharmacist is controversial (Moorkens et al., 2020a) because it compromises the physician’s prescribing autonomy, product traceability from a pharmacovigilance perspective, and existing market share agreements (Afzali et al., 2021). Percentage markups imply that pharmacists earn more money by dispensing more expensive biologics rather than biosimilars. Hence, implementing a system of regressive markups levels the playing field between originators and biosimilars. Few countries apply lower patient co-payments as an instrument to favour biosimilar dispensing. However, such a policy may be counteracted by other measures. For instance, countries such as Japan cap the total amount of co-payments that patients need to pay. This measure benefits higher-priced biologics over biosimilars, as the cap is reached faster with more expensive products.

#### 3.1.9 Monitoring

Monitoring of adverse events after commercialisation is a common requirement for all marketed drugs and is applied to biosimilars in nearly all selected countries (see Table 1). For instance, Norway has implemented a monitoring system that identifies different batches of the same product, with this information being included in a patient’s history upon switching or biosimilar treatment initiation (Davio, 2017). Monitoring can also provide a way of generating real-world evidence demonstrating the similar efficacy and safety of originator and biosimilar products. Transparency in both supply (from the manufacturer side) and usage (from the healthcare system side) ensures more predictability for all stakeholders and supports sustainable competition if additional manufacturers can be selected to compensate for any foreseen shortages [as is the case in the United Kingdom (KPMG, 2019)].

### 3.2 Sustainability rating of key biosimilar policy areas

For each of the nine key biosimilar policy areas defined, policies in each selected country have been rated according to their level of sustainability (see Table 2). Generally, current approaches to biosimilar manufacturing and R&D incentives, and exemptions to the application of health technology assessment to biosimilars are deemed to be sustainable by experts. Across countries, there is room for improvement with respect to biosimilar contracting approaches and with ensuring biosimilar education and understanding.

**TABLE 2:** Sustainability rating for each key biosimilar policy area in selected countries.

Policy area		AUS		BEL		BRA		CAN		FRA			DEU	GBR	ITA	JPN		
Manufacturing and R&D		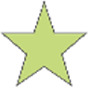		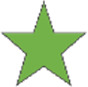		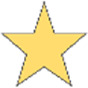		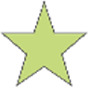		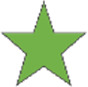			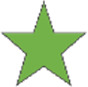	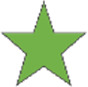	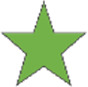	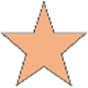		
Regulatory approval		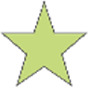		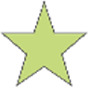		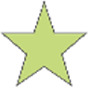		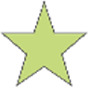		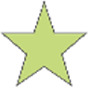			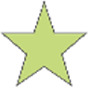	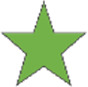	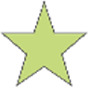	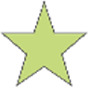		
Health technology assessment		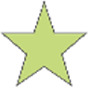		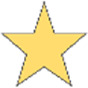		Priv. and Pub. 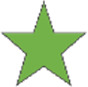	Pub. HC 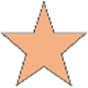	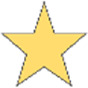		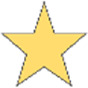			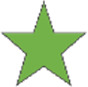	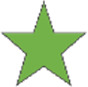	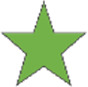	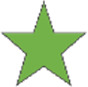		
Pricing and reimbursement		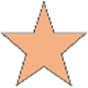		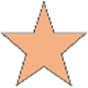		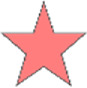		Pub. 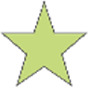	Priv. 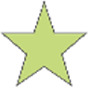	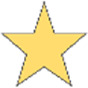			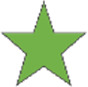	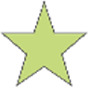	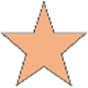	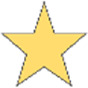		
Contracting		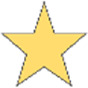		Inp. 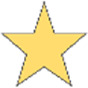	Out. 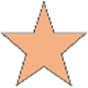	Pub. 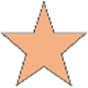	Priv. 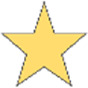	Pub. 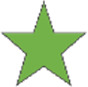	Priv. 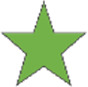	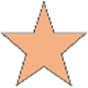			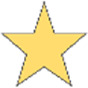	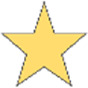	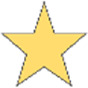	N/A		
Biosimilar education and understanding		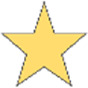		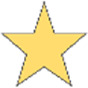		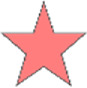		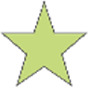		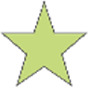			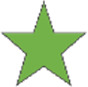	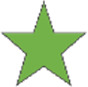	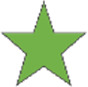	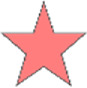		
Prescribing		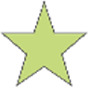		Inp. 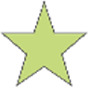	Out. 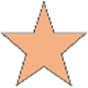	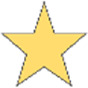		Pub. 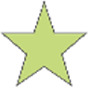	Priv. 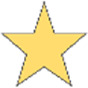	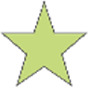			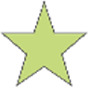	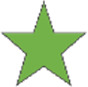	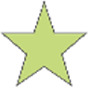	Inp. 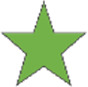		Out. 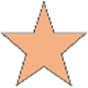
Dispensing		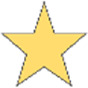		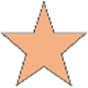		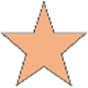		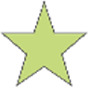		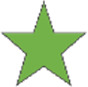			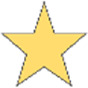	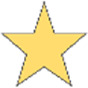	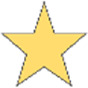	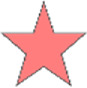		
Monitoring		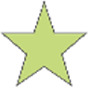		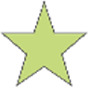		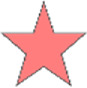		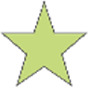		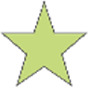			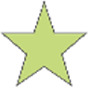	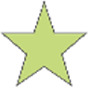	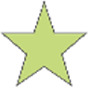	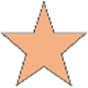		
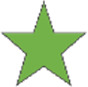	The policy area is considered to be sustainable for all stakeholders	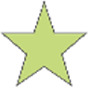	Some minor areas for improvement were identified to result in a fully sustainable environment; however, no unsustainable policies impact the area								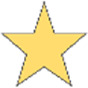	Some major areas for improvement were identified to result in a fully sustainable environment; however, no unsustainable policies impact the area						

(Continued on following page)

European countries, which tend to have more experience with biosimilar products and more developed policy, generally have higher sustainability scores. Key successes include high levels of uptake driven by acceptance and trust from physicians and patients, efficient access due to streamlined manufacturing, regulatory and health technology assessment approaches. Conversely, markets with more limited experience with biosimilars (e.g., Saudi Arabia, Japan) have less developed biosimilar policy, resulting in higher risks to market sustainability. Key challenges include minimal differentiation between biosimilar and generic policies, decreased traceability in pharmacovigilance systems, and high levels of mistrust in biosimilars based on miseducation or limited clarity in regulatory processes within the market.

In general, the assessment is more nuanced, varying by patient setting and type of product. It is often the case that policies that are sustainable for biosimilars dispensed in the inpatient setting are unsustainable when considering outpatient medicines. For example, Japanese prescribing policy is significantly more sustainable in the inpatient setting given the role of indirect incentives, which promote biosimilar use in a manner not seen in the outpatient setting.

Finally, based on the biosimilar sustainability ratings in these countries, best practice examples have been identified for each key policy area, some of which are reported in Table 3.

**TABLE 3:** Examples of best practices in selected countries by key biosimilar policy area.

Policy area	Best practice examples
Manufacturing and R&D	European Union legislation streamlines preparation for biosimilar entry prior to the loss of exclusivity, enabling rapid launch post patent-expiry
Regulatory approval	The United Kingdom Medicines and Healthcare products Regulatory Agency no longer requires clinical comparability studies for all products given latest research regarding their lack of additional value to regulatory assessments
Health technology assessment	Many countries like the Netherlands waive the need for biosimilar health technology assessment provided the indications included in the biosimilar label are the same as the originator
Pricing and reimbursement	In the Netherlands, biosimilars can launch at the same price as their originators, encouraging entry, then competition is used to promote cost savings. Moreover, pricing and reimbursement are applied as a single process, ensuring biosimilars’ automatic reimbursement
Contracting	In the United Kingdom, long-term supply plurality is provided for adalimumab biosimilars, given that the market has been divided into 11 hospital groups. These groups are allocated a specific biosimilar or originator product, with degressive market shares for those products dependent on the competitiveness of the tender price they have offered
Biosimilar education and understanding	European educational campaigns spearheaded by the European Medicines Agency are often supplemented with national-level education in European countries, for example, at hospital/provider level to ensure holistic understanding of value across the country
Prescribing	In the United Kingdom, non-mandatory prescribing quotas still serve as an incentive for healthcare professionals. Moreover, gainsharing mechanisms implemented at some local Clinical Commissioning Groups have ensured that savings driven by biosimilars are reinvested in healthcare systems, improving their perception
Dispensing	In France, current dispensing policies do not undermine physicians’ autonomy but instead promote shared decision-making also with pharmacists. Moreover, substitution policies do not interfere with robust tracing systems used for safety monitoring (i.e. 95% of retail pharmacies are connected to the traceability tool - the “dossier pharmaceutique” - even though not all of them use it systematically), and patients can have their voices heard without any misconceptions around biosimilar value being able to influence dispensing decisions
Monitoring	The United States has pharmacovigilance systems ensuring full transparency in monitoring, for example, by assigning a suffix to the biosimilar name in regulatory documents to distinguish between different biosimilars

### 3.3 Is there a general biosimilar policy toolkit that ensures sustainability?

This international experience suggests that there are some areas for which there is consensus on sustainable biosimilar policies. For example, policies ensuring safe and high-quality biosimilars (e.g., post-commercialisation pharmacovigilance measures, although countries recognised that this went beyond biosimilars and would take years to fully implement in some markets) are consistently considered sustainable. Similarly, policies supporting mitigation of frequently held biosimilar misconceptions (e.g., multi-stakeholder educational programs led by patient advocacy groups and/or governmental organisations) are also considered prerequisites for sustainability.

However, there are instances where seemingly similar policies are applied in different markets and are seen as sustainable in some, but unsustainable in others. For example, use of healthcare professional incentives in the United Kingdom to drive initial uptake of biosimilars is seen as a means of improving sustainability. However, such measures have been highlighted as unsustainable in Latin American countries like Mexico, given the lack of transparency around them and the perception that this inappropriately influences physician behaviour. In other markets, incentives are seen as a temporary policy to boost initial biosimilar adoption that should slowly be removed as the community gains knowledge and experience with these products. Also, the sustainability of a given policy can vary depending on the type of biologic used for treatment. For example, incentivising switching to best alternative treatments can be seen as a positive regulation to increase biosimilar uptake in areas like oncology, where the focus is on initiation of new patients and treatment is of a shorter duration. However, there are different concerns for chronic diseases where patients will be on treatment for an extended duration, and patients and physicians may have preferences for a particular product.

More generally, the sustainability of biosimilar policies depends on the nature of the healthcare system and existing pharmaceutical policies (e.g., pricing and reimbursement processes, contracting approaches), the history and experience with biosimilar use, and the way that a biosimilar is prescribed or dispensed. This indicates that the biosimilar policy toolkit that ensures sustainability will vary from country to country and with the type of biologic losing protection. Therefore, based on the literature review, this study has instead defined a set of general elements that should underpin sustainable biosimilar policy development over time (see Table 4). Initially, biosimilar policies should focus on ensuring the safety and quality of biosimilars, safeguarding healthy levels of supply and delivering a level of cost savings. As biosimilars become more established, policies should seek to optimise uptake, and combat any misconceptions regarding biosimilars. Ultimately, countries should aim for biosimilar policies that encourage competition, broaden treatment options and ensure a sustainably functioning biosimilar market.

**TABLE 4:** Elements that should underpin sustainable biosimilar policy development over time.

Sustainable biosimilar policies should:			
INITIALLY AND AT A MINIMUM			
1	Ensure safe and high-quality medicines	Policies should ensure high quality medicines with robust and transparent evaluations, and monitoring systems to give confidence to patients and healthcare professionals	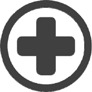
2	Facilitate cost savings for healthcare providers	Policies should facilitate cost savings for healthcare systems to ensure long-term budget sustainability	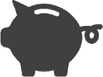
3	Ensure healthy levels of supply	Policies should minimise risks of supply shortage and ensure there is sufficient demand for biosimilars to avoid wastage or incentives to sell at unsustainable prices	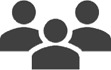
4	Maintain incentives for continued biologic research & innovation	Policies should ensure that sufficient incentives for manufacturers remain in place to ensure that there is continued research to launch new biologic products	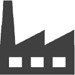
AS BIOSIMILAR POLICY MATURES			
5	Mitigate against biosimilar misconceptions	Policies should seek to address common concerns surrounding biosimilars to optimise uptake and ensure informed decision-making across all stakeholders	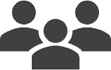
6	Facilitate efficient & streamlined patient access	Policies should encourage streamlined access procedures without compromising safety to ensure eligible patients have unrestricted access to life-saving medicines	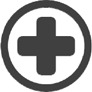
7	Encourage multi-stakeholder decision-making	Policies should ensure that all key stakeholders (payers, physicians, pharmacists and patients) play a role within decision-making to optimise multi-stakeholder benefits	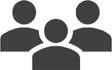
FINALLY TO ENSURE LONG-TERM SUSTAINABILITY			
8	Facilitate sustainable levels of biosimilar competition	Policies should ensure that market competition is incentivised to ensure long-term predictable price levels, while delivering savings that may be reinvested	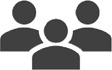
9	Increase prescribing options for patients & healthcare professionals	Policies should encourage availability of multiple prescribing options to maintain flexibility in treatment regimens to address individualised patient needs	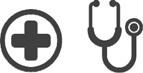
10	Maintain predictable market functioning	Policies should ensure that market volatility is kept to a minimum and that policies are transparent to maintain attractivity of thes market	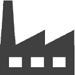
BENEFITS FOR: 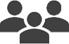 = All stakeholders 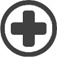 = Patient 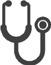 = Healthcare Professional 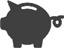 = Payers / Budget Holder 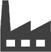 = Biosimilar & Originator Manufacturers			

### 3.4 Recommendations for biosimilar sustainability by key policy area

While sustainable biosimilar policies may vary from country to country, common themes and recommendations for each key policy area were identified that need to be followed with a view to ensure a sustainable biosimilar environment.

#### 3.4.1 Manufacturing and R&D

Biosimilar manufacturing policies should ensure the highest standard of quality and allow for prompt submission to regulatory authorities upon loss of exclusivities of the originator biologic while respecting intellectual property. Countries that have previously faced supply issues (e.g., Brazil) could provide incentives for sustainable local manufacturing to reduce shortages, provided that competition is encouraged and as long as they do not penalise biosimilars manufactured in other countries, so as not to disrupt global supply chains.

#### 3.4.2 Regulatory approval

Biosimilar regulatory processes should seek efficiencies to accelerate access timelines, while maintaining robust processes that ensure quality, efficacy and safety of biosimilars. Regulators should consider the biosimilar type, number of biosimilars already available and the requested indication to determine required evidence for submission. With respect to evidence requirements on efficacy, regulatory agencies should adopt the latest scientific consensus regarding the need and value of comparative clinical trials. Regulatory processes should be consistent and transparent across global markets, aiding countries with smaller regulatory agencies (e.g., Saudi Arabia or the United Arab Emirates) to leverage experience of agencies with more capacity.

#### 3.4.3 Health technology assessment

Conventional health technology assessment is not necessary given that the therapeutic benefit provided by a biosimilar is similar to that of the originator. However, it is warranted when, for example, the originator biologic is not reimbursed, the biosimilar offers a different route of administration than the originator, or the biosimilar provides value-added services compared to the originator (Moorkens et al., 2020b). In these cases, the assessment provides tangible benefits such as the ability to differentiate between products within tenders.

#### 3.4.4 Pricing and reimbursement

Depending on the policy landscape, either mandatory price controls or dynamic price controls (reliant on market competition) can be implemented provided that there are safeguards to ensure competitive, but sustainable price levels. When mandatory discounts are applied (e.g., in France, Spain and Italy), policies should recognise that a one-size-fits-all approach for biosimilars may not be sustainable in the long term and consider differences across therapeutic areas, the number of competitors and population size. When dynamic price controls are applied (e.g., in the Netherlands), policies should safeguard multiple market participants to ensure sufficient levels of competition are maintained.

Automatic reimbursement systems which operate in a transparent manner (e.g., as seen in Germany) support more efficient access for biosimilars. When automatic reimbursement is not used, measures to streamline pricing and reimbursement pathways should be considered such as early initiation of negotiations (e.g., as done by the pan-Canadian Pharmaceutical Alliance) or acceleration of existing processes (e.g., as done in Belgium).

#### 3.4.5 Contracting

Contracts can be awarded through tendering or direct negotiation. When tendering processes are the primary procurement method (e.g., in Spain and the United Kingdom), award criteria should extend beyond price and consider elements of value (e.g., value-added services) and ability to supply. Transparent, equal opportunity must be granted to all suppliers with the appropriate and consistent application of award criteria that contribute meaningfully to final contractual decisions. When direct contracting is the main procurement method (e.g., in the United States and the Belgian outpatient setting), exclusionary contracts should be avoided, since this could result in limited biosimilar competition (cfr. supra).

Competition should be preserved by safeguarding multiple participants. This implies that single-winner contracts at the national level should be avoided. Or when single-winner contracts operate regionally/locally, measures should be in place to ensure multiple suppliers at national level. Supply issues and withdrawal due to unsustainable revenues should be avoided by payers imposing appropriate enforcement measures on suppliers and by providing market share guarantees to individual suppliers over a set period of time, respectively. With respect to contract duration, longer tenders are required for those therapy areas where longer treatment periods are expected, such as for chronic diseases, so that patients may have to switch treatment less frequently.

#### 3.4.6 Biosimilar education and understanding

All key stakeholders (e.g., policymakers, payers, healthcare professionals and patients) should be educated on the benefits of biosimilars. Biosimilar advocates should work together in a multidisciplinary manner to ensure that messaging across all channels is consistent. Educational materials should be independently developed (from a credible source) in order to support evidence-based education. In countries where there is greater biosimilar uptake (e.g., the United Kingdom), the experience of physicians can be shared with physicians and patients in specialities newer to biosimilars. In countries where there are persistent biosimilar misconceptions (e.g., Japan and Brazil), education of the most influential stakeholders (e.g., policymakers, key opinion leaders) should be a priority to ensure biosimilar uptake.

#### 3.4.7 Prescribing

The physician is responsible for ensuring that the most appropriate biologic is prescribed to each individual patient. It is crucial that prescribing decisions are informed by appropriate education of the benefits of biosimilars not only for the healthcare system as a whole, but also for individual patients. Prescribing incentives can stimulate initial uptake, but may be withdrawn as prescriber education and experience improves over time. Direct financial incentives to healthcare professionals can be seen as unsustainable when there is a lack of transparency (as is the case in, for example, Mexico). However, an indirect financial incentive such as gainsharing can provide a holistic approach which ensures that physicians, patients and healthcare systems all benefit from the savings driven by biosimilars.

Prescribing by international non-proprietary name can reduce originator bias by equalizing perception across originator and biosimilars. However, the nomenclature employed must allow for a correct differentiation of molecules (e.g., batch number, unique identifiers) to keep accurate monitoring and traceability upon switching in order to maintain a sustainable pharmacovigilance system (if other provisions do not establish this already).

#### 3.4.8 Dispensing

There is a debate regarding the role of substitution in many countries. Any decision should be based on multidisciplinary input to ensure the best outcomes for patients and the best value for the healthcare system. It should be recognized that no “one size fits all” approach will work while there is variation in available switching data, setting of care (inpatient vs. outpatient) and individual therapies. Financial incentives for pharmacists should not penalise them for dispensing biosimilars (e.g., through lower margins for lower-cost products) and should be aligned to the incentives in place for physicians. In markets where outpatient substitution is used (e.g., Brazil and Mexico), measures to minimise friction between pharmacists and physicians should be implemented, for example, by means of physician notification. Efforts should be made to share experiences with substitution schemes between countries.

#### 3.4.9 Monitoring

Biosimilars should be subject to the same pharmacovigilance standards as all biologics. Stakeholder-led monitoring should be encouraged, with physicians, pharmacists and patients being empowered and educated to report adverse events through a simple process. Various measures to maximise traceability can be implemented, including electronic systems that record individual patient prescriptions and the product used to fill each prescription; notification systems that ensure that any prescription changes are shared with the prescribing physician for approval/notification; or automated systems that facilitate easy identification of patients who have been dispensed medicines flagged for recall or additional safety follow-up.

## 4 Conclusion

Even though the implementation of sustainable policies is specific to each country, the following overarching learnings emerged from our comparative analysis of the sustainability of biosimilar policies in 17 countries worldwide:

• The introduction of biosimilar policy should be anchored in supporting the goal of sustainability in the short and medium term, ensuring cross-stakeholder perspectives are captured.
• As a country’s biosimilar landscape matures over time and stakeholder experience increases, there is a need to periodically evaluate and update policies to ensure sustainability is maintained.
• Policies are less effective when implemented in a piecemeal fashion, hence implementation should consider the existing policy environment and leverage synergies across policy areas.
• Cultivation of a sustainable global biosimilar landscape requires sharing of learning and best practices across markets, to support accelerated development in countries with less mature biosimilar landscapes.

To complement our analysis, future research could adopt a quantitative approach to assessing biosimilar sustainability and measure how a biosimilar market performs in terms of various sustainability indicators such as price erosion over time, the number of competitors and the occurrence of shortages. Decision makers can then consider relevant policy measures depending on the sustainability level of the biosimilar market.

## Data Availability

The original contributions presented in the study are included in the article/supplementary material, further inquiries can be directed to the corresponding author.
